# Immature Stages and Breeding Behaviour of the Hollyhock Weevil–*Rhopalapion longirostre* (Olivier 1807) (Coleoptera, Brentidae, Apioninae)

**DOI:** 10.3390/insects16020143

**Published:** 2025-02-01

**Authors:** Rafał Gosik

**Affiliations:** Department of Zoology and Nature Protection, Maria Curie–Skłodowska University, Akademicka 19, 20-033 Lublin, Poland; r.gosik@poczta.umcs.lublin.pl

**Keywords:** *Rhopalapion longirostre*, biology, morphology, development, sexual behaviour, *Pexicopia malvella*

## Abstract

Since the 1960s, the range of *Rhopalapion longirostre* has rapidly expanded from central Asia to almost all of Europe; it has also been introduced to North America. This expansion is an expression of this weevil’s response to climate change. The phenology of *Rhopalapion* has been comprehensively described. Its feeding and development are associated with plants from the genus *Alcea*. Even though more than 300 scientific papers have been written on various aspects of *R. longirostre*, its immatures and certain kinds of behaviours have yet to be described. The aims of this study were (1) to provide new information on the biology of this species, in particular with regard to the morphology of the preimaginal stages, and (2) to present new observations of its reproductive and developmental behaviour. A total of 73 larvae and 67 pupae were collected from 20 hollyhock plants. Of these, 21 mature larvae and 42 pupae (21 male and 21 female) were measured and described morphologically. The descriptions of both larvae and pupae revealed a number of original features, not previously observed on known immatures of Brentidae. The sympatric development of caterpillars of *Pexicopia malvella* (Hübner, (1805)) in hollyhock fruit was recorded as a pathogenic factor limiting the numbers of *R. longirostre*.

## Simple Summary

New observations on the reproductive behaviour of hollyhock weevil–*Rhopalapion longirostre* (Olivier, 1807) are presented. The morphology of the preimaginal stages of this species is comprehensively described for the first time. The pupal measurements indicate the existence of two morphological forms: a very considerable number of relatively large individuals (body length: 3.00–3.50 mm) and a few very small (1.75, 2.30 mm) individuals. Competition between *R. longirostre* and the hollyhock seed moth—*Pexicopia malvella* (Hübner, (1805))—for the development niche is described. New information is given on the distribution of *R. longirostre* as well as evidence confirming the connection between global warming and its expansion.

## 1. Introduction

The genus *Rhopalapion* Schilsky, 1906 contains only two species: *R. celatum* Giusto, 2021a and *R. longirostre* (Olivier, 1807) [1]. The bilaterally flattened, elongate body, elongate antennal club and elongate rostrum in females make the genus readily distinguishable within the family Brentidae [1,2].

The range of *R. celatum* is restricted to central Asia. Since the 1960s, *R. longirostre* has rapidly expanded its range from central Asia to the Middle East and the Mediterranean region, across the whole of Europe (except Scandinavia) to the British Isles. Moreover, *R. longirostre* was introduced to North America, where it has since spread from East to West. In mountain regions, it has been recorded at altitudes up to 2150 m a.s.l. [1,2,3,4,5,6]. According to Knutelski and Petryszak [7] and Sprick et al. [8], the north-westward expansion of *R. longirostre* appears to be a response to climate change. At the same time, its patchy distribution, particularly within residential areas, clearly indicates that its expansion is anthropogenic. It is worth mentioning that these beetles, especially the females, are quite efficient, active fliers [9].

Both *Rhopalapion* species are heliophilic, xerothermophilous weevils inhabiting pastures, meadows, roadsides, ruderal communities, and gardens, especially in Europe. That is why in some countries the weevil is regarded as a pest of ornamental plants [10,11].

Feeding and development in the genus *Rhopalapion* is closely associated with plant species like *Alcea digitata* Alef., *A. dissecta* (Baker f.) Zohary, *A. rosea* L. and *A. setosa* (Boiss.) Alef. Current observations do not endorse historical records regarding its occurrence on other genera in Malvaceae or on many other plant families. Moreover, records of *R. longirostre* on cotton plants (*Gossypium* sp.) in Turkey and the USA require confirmation [1].

The phenology of *Rhopalapion* is well known, having been summarized in detail by Giusto [1]. The adults are active (depending on the latitude) from March to November, but under certain conditions, they may be active all year round. Oviposition takes place from May to July, with females laying eggs in holes bored in the flower buds. After 3–4 days, the first instar larva hatches, migrates to the ovary and enters a single pericarp, where it starts to consume the seed, forming a chamber inside the pericarp. Then, it bores an escape hole in the side of the pericarp, which is subsequently sealed with a protective secretion. Pupation takes place inside the pericarp and lasts 2–6 weeks, depending on the weather conditions. The whole developmental period lasts for 7–10 weeks. Because of the species’ prolonged period of activity, different generations may coexist during a single season. Overwintering takes place under plant remains close to the host plant [12,13,14,15,16,17]. Being the subject of over 300 publications [1], *R. longirostre* has become one of the most frequently chosen models in morphological [18], ecological and zoogeographical studies [19].

The aims of this study were (1) to provide new information on the biology of this species, in particular with regard to the morphology of the preimaginal stages, and (2) to present new observations of its reproductive and developmental behaviour.

## 2. Materials and Methods

Larvae: 21 exx, L3, 22 July 2024, Lublin, Poland, garden, from hollyhock seeds.

Pupae: 42 exx: (10♀, 10♂), 24 July 2024, Prawiedniki, Poland, side road, from hollyhock seeds; (11♀, 11♂), 31 July 2024, Lublin, Poland, garden, from hollyhock seeds.

The activity, behaviour, and breeding of this species were observed on many hollyhocks growing in gardens, side roads and ruderal plant communities in Lublin (Poland) and Zemun (Serbia) areas during the 2023 and 2024 seasons. The immature stages for the morphological description were collected in Lublin.

Five to ten schizocarps were collected from each of the 20 hollyhock exemplars. Breeding was carried out according to the method developed by Toševski [20]. Ultimately, 73 larvae and 67 pupae were obtained, from which 21 mature larvae and 42 pupae (21 male and 21 female) were measured and described morphologically.

All the laboratory experiments and breeding took place at the Maria Curie-Skłodowska University in Lublin. Before writing the description, all the specimens were fixed in 75% ethanol and examined under an optical stereomicroscope (Olympus SZ 60 and SZ11) with calibrated oculars. The following measurements of the larva were taken: body length (BL), body width (BW) (at the third thoracic segment), head capsule width (HW) and head capsule height (HH, measured from the apex to the epistoma). The pupal measurements included body length (BL), body width (BW) (at the level of the mid-legs), head width (HW) (at the level of the eyes), rostrum length (RL) and pronotum width (PW). Slide preparation basically followed May [21]. The head of the larva selected for microscopic study was cut off and cleared, after which the mouthparts were separated. The remaining part of the body was cleared in 10% potassium hydroxide (KOH), then rinsed in distilled water and dissected. Thereafter, the head, mouthparts and body (thoracic and abdominal segments) were separated and mounted on permanent microscope slides in Faure-Berlese fluid (50 g gum arabic and 45 g chloral hydrate dissolved in 80 g distilled water and 60 cm^3^ glycerol) [22].

The photographs were taken usings an Olympus BX63 microscope and processed with Olympus cellSens Dimension software (version 1.18). The larvae selected for SEM (scanning electron microscope) imaging were first dried in absolute ethanol (99.8%), then rinsed in acetone, treated by CPD (Critical Point Drying) and finally gold-plated. TESCAN Vega 3 SEM was used to examine selected structures.

The general terminology and chaetotaxy follow Anderson [23], May [21], Marvaldi [24,25] and Trnka et al. [26]; the terminology for the antennae follows Zacharuk [27].

## 3. Results

### 3.1. Morphology

#### 3.1.1. Description of the Mature Larva

**Figure 1:**
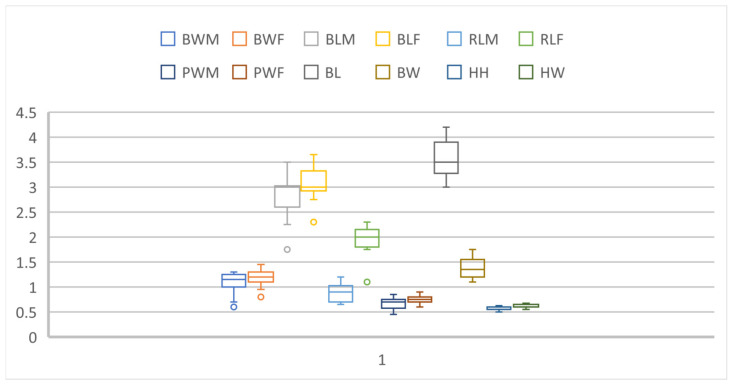
Comparison of sizes of selected body parts of pupae and mature larvae of *Rhopalapion longirostre*. M, male; F, female; BL, body length; BW, body width; HH, head height; HW, head width; PW, pronotum width; RL, rostrum length. All measurements are in millimetres (mm).

All the measurements are given in Table 1 and Figure 1.

Mean values: male: BL–3.59, BW–1.36, HH–0.57, HW–0.62.

**Figure 2:**
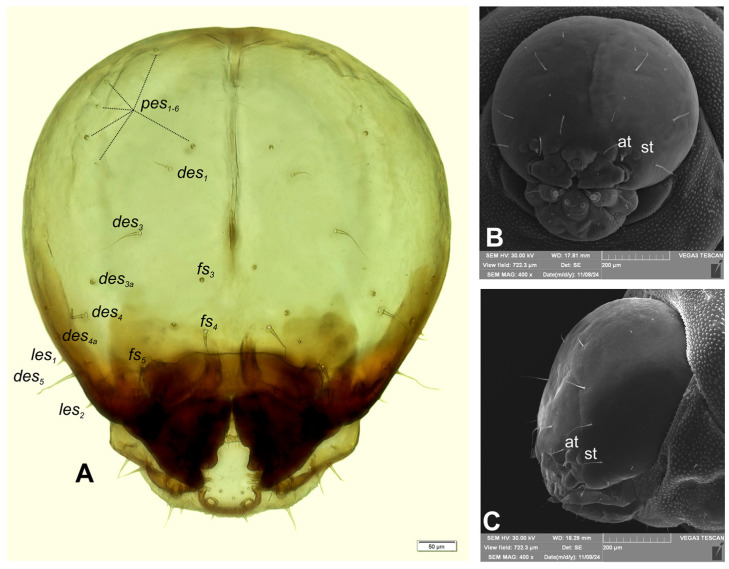
*Rhopalapion longirostre* mature larva, head: (**A**)—frontal view (photo); (**B**)—frontal view, (**C**)—lateral view (SEM micrograph) (at—antenna, st—stemmata, setae: *des*—dorsal epicranial, *fs*—frontal, *les*—lateral epicranial, *pes*—postepicranial).

Head capsule (Figure 2A–C) perfectly rounded; endocarina reaches half-length of the frons; frontal sutures distinct along entire length up to antennae; stemmata (st) conspicuous, each placed to the side of the antennae. Setae of head hair–like, various in length: from elongate and medium to minute. Cranial setae: *des_1_* short, placed close to sutura coronalis; *des_2_* absent, *des_3_* elongate, placed above frontal suture; *des_4_* elongate, placed laterally; *des_5_* elongate, placed anterolaterally. Frontal setae: *fs_1_* and *fs_2_* absent; *fs_3_* minute, placed medially; *fs_4_* elongate, placed anteromedially; *fs_5_* elongate, placed anterolaterally, close to antenna; both *les_1_* and *les_2_* of equal length, very short; ventral setae absent; postepicranial area with 6 min *pes*. Pores present: one between *des_3_* and *des_4_*, one between *des_4_* and *des_5_*, and two between *fs_4_* and *fs_5_*.

**Figure 3:**
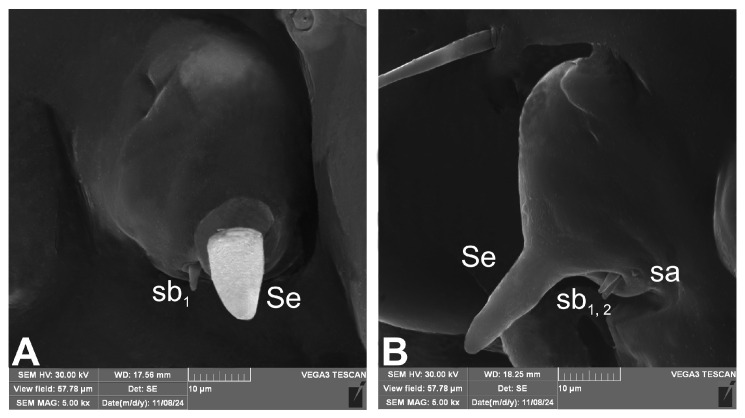
*Rhopalapion longirostre* mature larva, antenna (SEM micrograph): (**A**)—dorsal view, (**B**)—lateral view (sa—sensillum ampullaceum, Se—sensorium, sb—sensillum basiconicum).

Antennae (Figure 3A,B) situated on each side of anterior margin of head; membranous basal segment convex, semi-spherical, bearing conical, elongate sensorium and two basiconica (sb) and single ampullaceum (sa).

**Figure 4:**
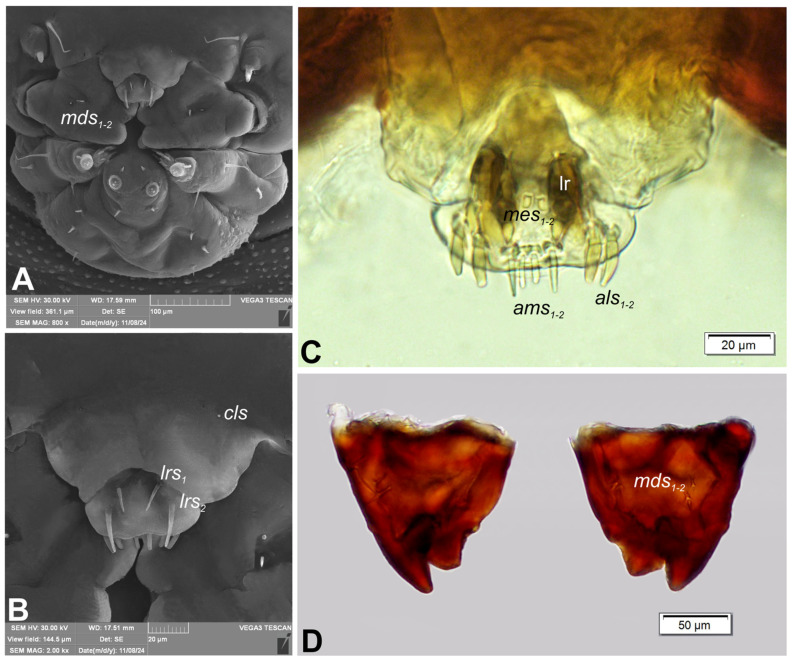
*Rhopalapion longirostre* mature larva, mouthparts: (**A**)—total, frontal view (SEM micrograph); (**B**)—clypeus and labrum, dorsal view (SEM micrograph); (**C**)—epipharynx, (photo); (**D**)—mandibles (photo) (lr—labral rods, setae: *als*—anterolateral, *mds*—mandibular, *ams*—anteromedial, *cls*—clypeal, *lrs*—labral, *mes*—median).

**Figure 5:**
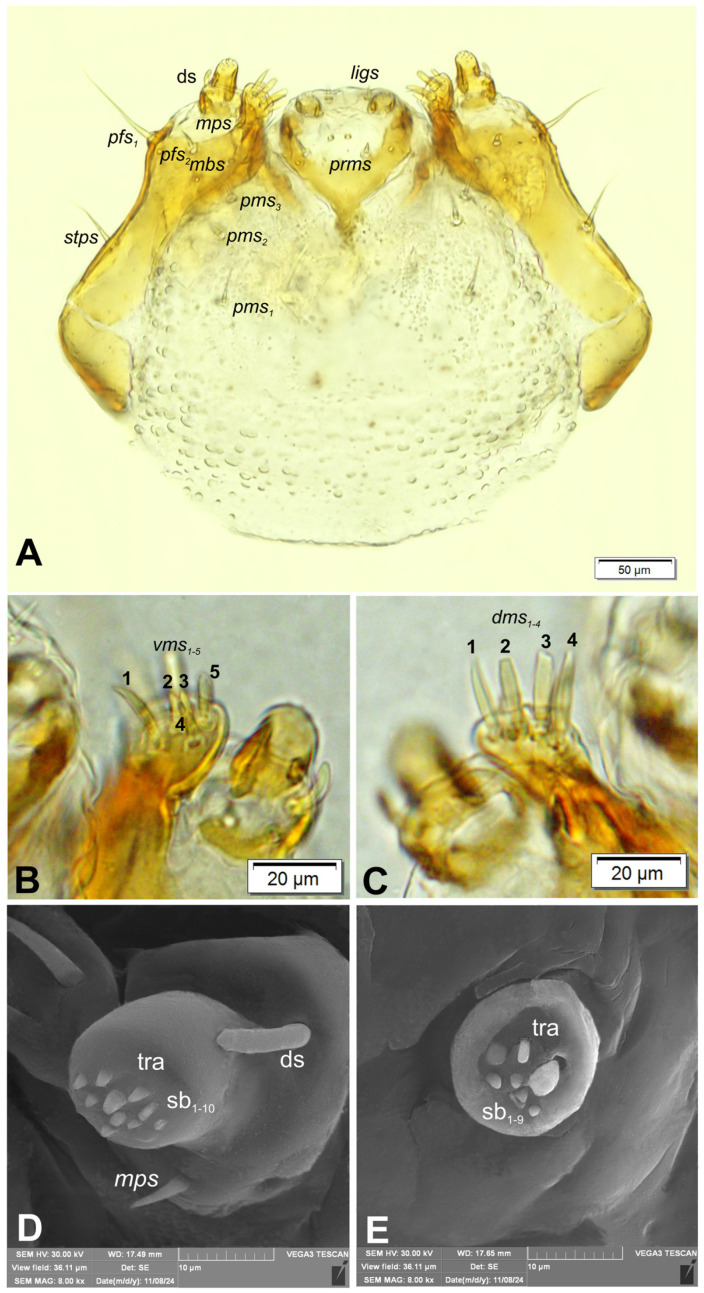
*Rhopalapion longirostre* mature larva, maxillolabial complex: (**A**)—maxillolabial complex, ventral aspect (photo); (**B**)—apical part of right maxilla, ventral aspect (photo); (**C**)—apical part of right maxilla, dorsal aspect (photo); (**C**)—maxillary palpomere (SEM micrograph); (**D**)—distal, labial palpomere; (**E**)—labial palpomere (SEM micrograph) (setae: *dms*—dorsal malar, *ligs*—ligular, *mps*—maxillary, *pfs*—palpiferal, *prms*—prelabial, *pms*—postlabial, *stps*—stipal, *vms*—ventral malar palp; sensilla: ds—digitiform, sb—basiconicum; tra—terminal receptive area).

Clypeus (Figure 4A,B) approximately 2.6× wider than long, with single, minute *cls*, placed posterolaterally. Anterior margin of clypeus strongly rounded to inside. Labrum (Figure 4B) approximately 2.5× wider than long, anterior margin slightly sinuate; *lrs_1_* medium, placed anteromedially; *lrs_2_* elongate, placed anterolaterally; *lrs_3_* absent. Epipharynx (Figure 4C) with 2 *als*, various in length; two digitate *ams*, equal in size; two digitate *mes* (first medium, second very short). Labral rods (lr) elongate, well sclerotized, parallel. Mandibles (Figure 4D) symmetrical, each with two apical teeth of unequal height, inner one very robust, outer tooth curved and much higher than inner one. Molar area with small, medially placed, conical protuberance. Setae: *mds_1_* short, *mds_2_* minute, both placed medially, in elongated depression. Maxillolabial complex: (Figure 5A) stipes with one medium *stps*, *pfs_1_* elongate, *pfs_2_* short and single minute *mbs*; mala with row of four digitate, almost equally sized *dms*, five *vms*: first elongate, second and third short, fourth medium, fifth elongate (Figure 5B,C); maxillary palpi two segmented; basal palpomere much wider than distal one; length ratio of basal and distal palpomeres almost 1:1; basal palpomere with minute *mps*, one pore and one robust digitiform sensillum (ds), distal palpomere (Figure 5D) with group of 10 apical sensilla (all basiconicae) on terminal receptive area (tra); labium with cup-shaped prementum, with one short *prms* placed medially; ligula concave, with one medium *ligs;* premental sclerite Y-shaped; postmentum rounded, with three *pms* of various length: short *pms_1_*, placed posteriorly, medium *pms_2_*, placed mediolaterally, and short *pms_3_* placed medially (Figure 5A). Labial palpi (Figure 5E) one segmented; each palpus with single pore, and group of nine basiconicae apical sensilla of various size on terminal receptive area; surface of labium densely covered with knobby asperities.

**Figure 6:**
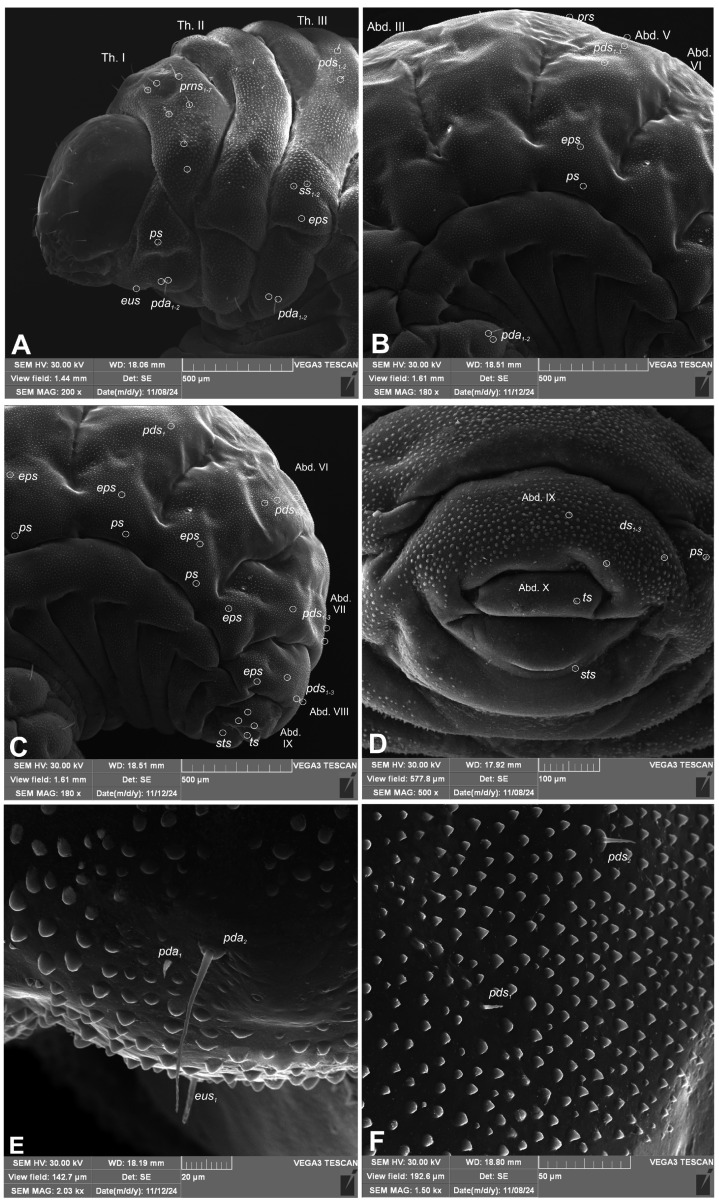
*Rhopalapion longirostre* mature larva, habitus and chaetotaxy (SEM micrographs): (**A**)—head and thorax; (**B**)—abdominal segments III–VI; (**C**)—abdominal segments V–IX; (**D**)—abdominal segments IX–X; (**E**)—pedal lobe; (**F**)—postdorsal lobe (setae: *ds*—dorsal, *ps*—pleural, *eps*—epipleural, *eus*—eusternal, *pda*—pedal, *pds*—postdorsal, *prns*—pronotal, *prs*—prodorsal, *ss*—spiracular, *sts*—sternal, *ts*—terminal).

**Figure 7:**
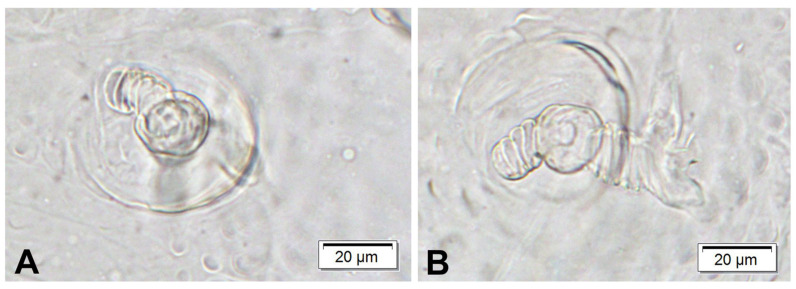
*Rhopalapion longirostre* larva, spiracles: (**A**)—s. of prothorax; (**B**)—s. of abd. I.

Live larva yellowish, with light yellow head capsule. Except for pronotal shield, whole body densely covered with knobby or conical cuticular processes. Body thick, strongly curved, rounded in cross section. Prothorax distinct, pronotal shield weakly separated, smooth. Mesothorax smaller than metathorax; each divided dorsally into two lobes (prodorsal and postdorsal lobes almost equal in size). Pedal lobes of thoracic segments weakly isolated, smooth. First abdominal segment as big as metathorax, segments II–V of similar size, much bigger than first abdominal segment; segments VI–IX tapering towards posterior end of body. Abdominal segments I–VII divided into two lobes of equal size. Abdominal segments VIII and IX dorsally undivided. Epipleural, laterosternal and eusternal lobes of segments I–VIII conical, well isolated. Abdominal segment X divided into two horizontally, external lobe slightly wider than inner one. Anus situated terminally. Except for pronotum, pedal area and terminal part of the abdomen almost invisible. Only a few thoracic setae, hair-like, moderately elongate, all other setae minute, hardly distinguishable from cuticular processes, sometimes absent. Thorax (Figure 6A): prothorax with seven medium-length, equally sized *prns* (three placed on premental shield, the others below), one *ps* and one *eus*, both short. Meso- and metathorax each with two medium *pds*, two min *ss* and one min *eps*. Pedal areas of thoracic segments each with two *pda* (first minute, second medium, both placed on isolated area) (Figure 6E). Abdomen (Figure 6B–D): segments I–VIII with one min *prs*, three *pds* (first minute, second and third short) (Figure 6F), one min *eps* and one min *ps* (segments VII and VIII without *ps*); segment IX with three short *ds*, one min *ps* and one min *sts*; external lobe of segment X with one min *ts* seta (Figure 6D). Spiracles (Figure 7A,B) on thorax unicameral, placed laterally between pro- and mesothorax; abdominal spiracles unicameral, placed anterolaterally on segments I–VIII.

#### 3.1.2. Description of Mature Pupa

All the measurements are given in Table 1 and Figure 1.

Mean walues: male: BW–1.09, BL–2.80, RL–0.90, PW–0.67; female: BW–1.19, BL–3.09, RL–1.93, PW–0.76.

**Figure 8:**
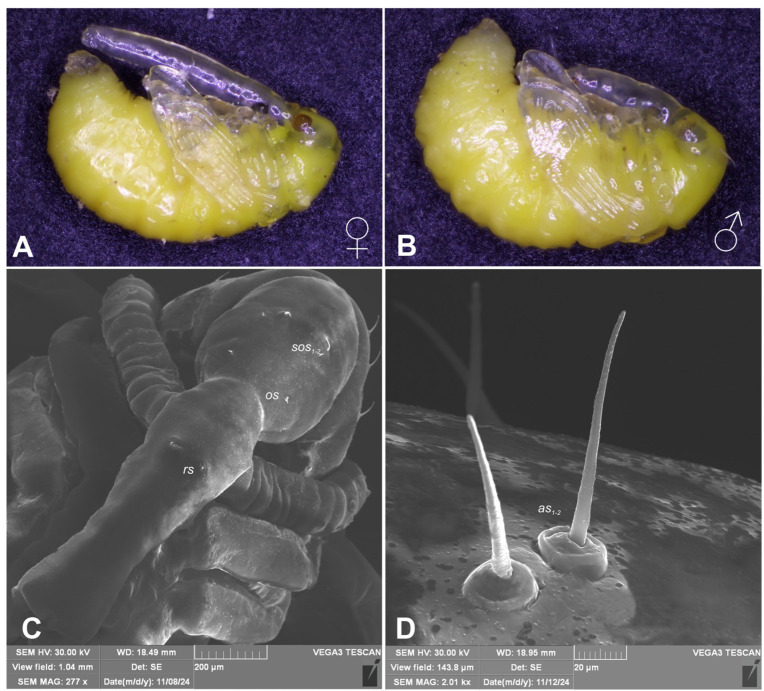

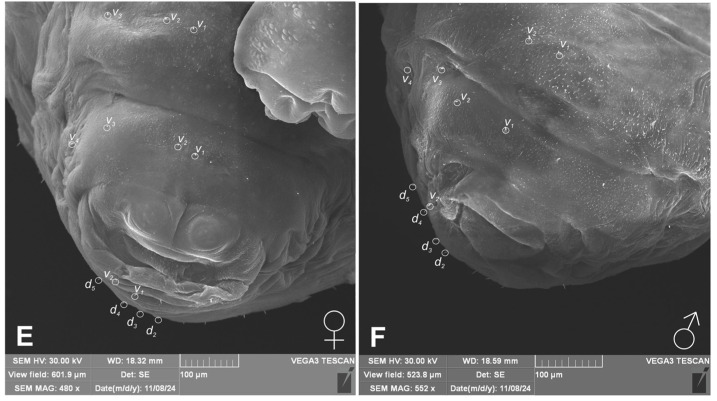
*Rhopalapion longirostre*: (**A**)—pupa, female; (**B**)—pupa, male; (**C**)—head and rostrum, dorsal view; (**D**)—pronotum, apical setae, magnification; (**E**)—female gonotheca, ventral view; (**F**)—male gonotheca, ventral view (setae: *as*—apical, *os*—orbital, *rs*—rostral, *sos*—superorbital, *v*—ventral).

Body yellowish, elongate, slightly curved. Cuticle on head, rostrum and thorax smooth, on abdomen irregularly covered with minute, hair–like asperities. Rostrum robust, longer than the whole body in female, reaching metacoxae in male (Figure 8A,B). Pronotum trapezoidal, slightly elongate. Mesonotum narrower than metanotum. Abdominal segments I–VII of equal length, segment VIII semicircular, segment IX terminal. Gonotheca undivided in male, divided in female, with tubercles on subcontiguous lobes. Urogomphi (posterior processes) absent (Figure 8E,F). Spiracles placed laterally on abdominal segments I–VI, functional on segments I–V, vestigial on segment VI. Clubs sparsely covered with conical protuberances.

**Figure 9:**
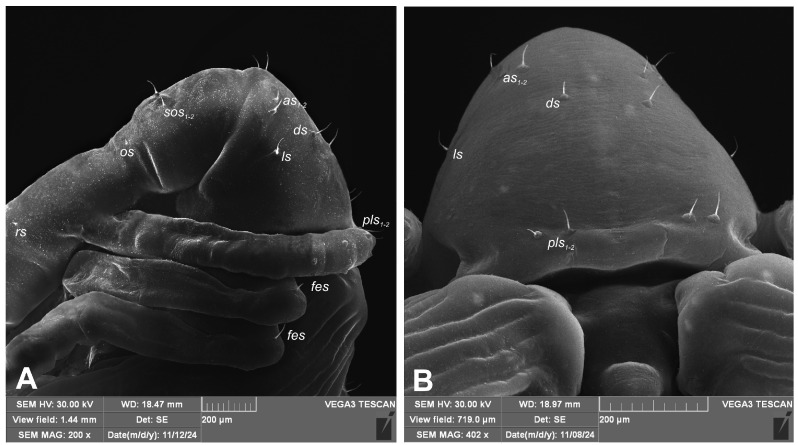

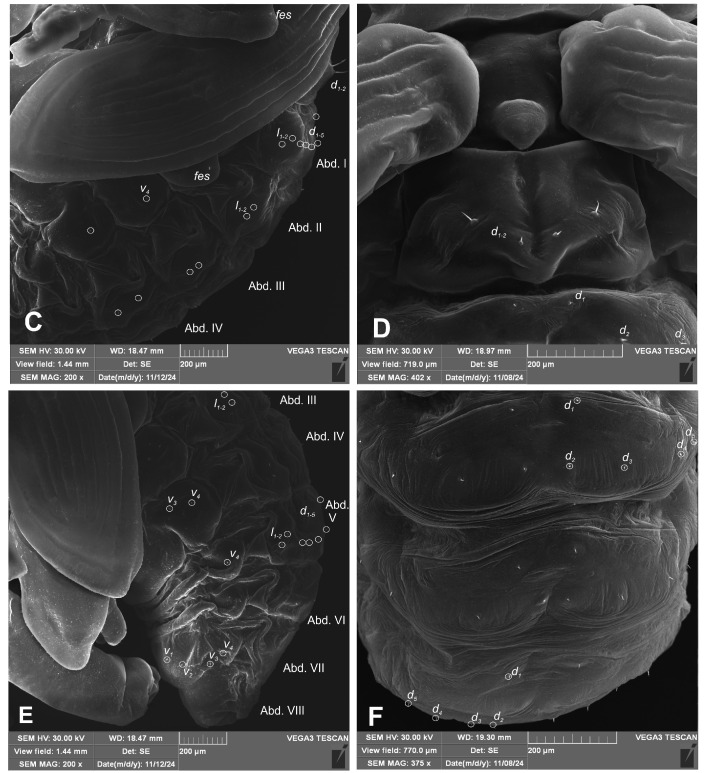
*Rhopalapion longirostre* pupa, (SEM): (**A**)—head and pronotum lateral view; (**B**)—pronotum, dorsal view; (**C**)—abdomen, lateral view; (**D**)—meso- and metanotum, dorsal view; (**E**)—abdomen, lateral view; (**F**)—abdomen, dorsal view (setae: *as*—apical, *d*—dorsal, *ds*—discal, *fes*—femoral, *l*—abdominal lateral, *ls*—thoracic lateral, *os*—orbital, *pls*—posterolateral, *rs*—rostral, *sos*—superorbital, *v*—ventral).

Chaetotaxy is easily seen only on head and pronotum, hardly visible on rest of body, discernible only under the highest magnification, sometimes absent. Head with one medium-length, one short *sos* and one minute *os*. Rostrum with single, minute *rs*. Pronotum with two *as*, one *ds*, one *ls* and two *pls*. All pronotal setae medium-length, equally long, on small protuberances (Figure 8B). Dorsal parts of mesothorax without setae; metathorax with one medium-length and one short setae, situated medially. Apex of femora with single, medium-length *fes*. Abdominal segments I–VIII with five minute setae dorsally (first placed posteromedially, second to fifth placed anteromedially). Each lateral part of abdominal segments I–VIII with two minute setae. Ventral parts of abdominal segments I–VIII with four minute setae (first and second placed medially, third and fourth lateromedially). Abdominal segment IX with two minute setae placed anteromedially (Figure 9A–F).

## 4. Discussion

**Figure 10:**
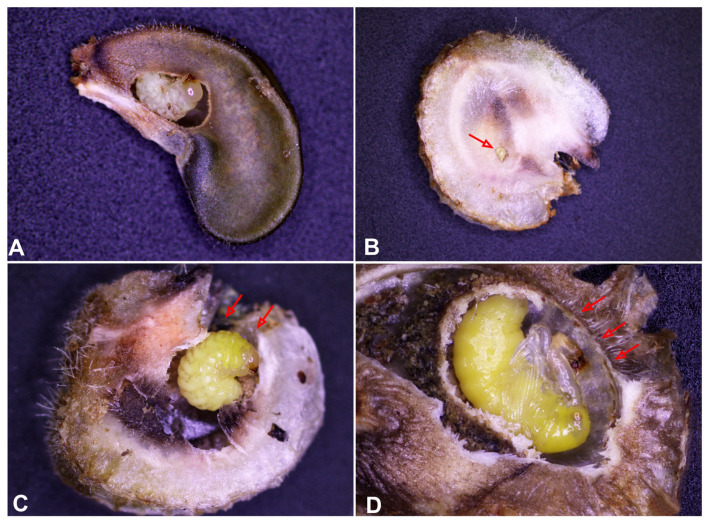
*Rhopalapion longirostre*: (**A**)—larva feeding in hollyhock seed; (**B**)—pericarp of hollyhock with hole bored by larva; (**C**)—mature larva in pericarp; (**D**)—pupa in pericarp (the red arrows indicate the exit holes).

*R. longirostre* is subject to evolutionary pressure promoting large-bodied individuals. In the case of females, the large body size (long snout) allows them to effectively drill a deep hole in the perianth of the corona and then lay the egg near the ovary [18]. Wilhelm et al. [28] state that only males of sufficient size have any chance of mating with large females. Hence, large-bodied individuals are favoured in both sexes (Figure 1). According to Wilhelm et al. [17], *R. longirostre* and *Alcea* sp. are excellent examples of the competition between an animal and its host plant, as described by Toju and Sota [29]. On the other hand, both Pupier [14] and Wilhelm et al. [18] note that approximately 15% of teneral adults fail to escape from the pericarp after pupation and ultimately die, there being more females than males among the individuals stuck inside the pupal cradles. Pupier [14] states that the adult escapes from the pericarp by enlarging the exit hole previously bored by the larva. Paradoxically, the smaller the individual, the greater its chances of successfully exiting the pupal cradle (Figure 10A–D). Since the average widths of the pupal pronotum are 0.70 mm (male) and 0.75 mm (female) and are slightly smaller than the diameter of the seed (on average 0.85 mm at its widest point), in practice, only the smallest specimens are able to rotate freely inside the pupal cradle. In females, the long snout additionally impairs the ability to manoeuvre freely. In practice, the effective lowering of the pericarp depends on how large a hole the larva bored and what position the larva took up before pupation. Tenerals have the best chance of leaving the pericarp when the exit opening is located near the abdomen in relation to the position of the pupa. However, if it is situated on the dorsal side of the pupa or near the head, the chances of its enlargement and the subsequent exit of the teneral beetle are small.

**Figure 11:**
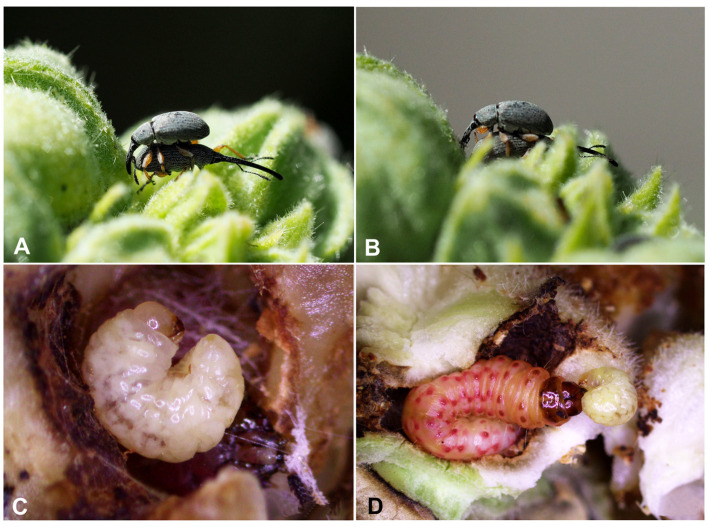
(**A**,**B**)—*Rhopalapion longirostre* pairs, males in reverse position; (**C,D**)—larva of *R. longirostre* expelled from the pericarp by a larva of the hollyhock seed moth (photos R. Gosik).

Wilhelm et al. [18] give an accurate description of the selection mechanisms among pairs for copulation in *R. longirostre*. It is worth adding, however, that in the life cycle of *R. longirostre*, there is quite a long period between the onset of activity (early March) and the period of breeding and oviposition (May). During this time, males can be observed adopting a reverse position upon the female (Figure 11A,B). This allows them to “occupy” her before she is ready to mate and at the same time enables them to actively repel other males without the risk of being pushed away.

The fieldwork yielded no parasitoids or pathogens that eliminated *R. longirostre* specimens during the preimaginal period. Although Wilhelm et al. [18] mention that larvae and pupae of *R. longirostre* are attacked by fungi or mites, it seems that their presence is secondary and occurs only after the death of the insects. However, one pathogenic factor limiting the number of *R. longirostre* is the sympatric development of caterpillars of a moth *Pexicopia malvella* (Hübner, (1805)) in mallow fruits.

Even though the weevil larvae are not a food resource for the caterpillars (no weevil larvae being eaten by this moth’s caterpillars have been observed), both species compete for the same ecological niche. Whereas one hollyhock seed provides sufficient resources for the development of one weevil, the moth caterpillar eats all the seeds in a schizocarp one after another. The caterpillar gnaws through all the seeds one by one, and on encountering weevil larvae or pupae, it either destroys them mechanically or bites a hole in the pericarp and expels the immature weevils (Figure 11C,D). Then, the exit hole is sealed with thread. In this way, one caterpillar can effectively prevent the development of 30 and more weevils. The hollyhock seed moth begins oviposition in mid-June [30]. Because the weevil starts laying eggs earlier than the moth, the latter has no opportunity to choose unoccupied fruit.

To date, within the subtribe Malvapiina Alonso-Zarazaga, 1990, larvae of only one species other than *R. longirostre* have been described, i.e., *Malvapion malvae* (Fabricius, 1775) [31], and no other pupae. Hence, isolating the characteristics of the group is impossible at this stage of research. Additionally, the development of *Malvapion malva* (Fabricius, 1775) on hollyhock requires confirmation. Unlike *R*. *longirostre*, each larva of other Malvapiina empties two to three seeds during its growth. This is probably why they never form populations as large as *R. longirostre* and are generally considered rare [32,33,34].

Wang et al. [35] first drew attention to the primary asymmetry of the mandibles in the larvae of *Pseudoaspidapion botanicum* Alonso–Zarazaga and Wang, 2011. *Diplapion confluens* (Kirby, 1808) also has asymmetrical mandibles, a fact that was omitted in the original description [36].

Chaika and Tomkovich [37] noted that polyphagous larvae have a greater diversity of sensilla than monophagous larvae. The fact that the monophagous larva of *R. longirostre* is equipped almost exclusively with basiconic sensilla fully endorses this observation. 

In addition to several other weevils that develop on hollyhocks, e.g., *Aspidapion aeneum* (Fabricius, 1775) or *Alocentron curvirostre* (Gyllenhal, 1833), *R. longirostre* is another species which is rapidly expanding northwards [38,39]. In some countries, this gives it the status of an alien, invasive or even pest species [40]. The main factors enabling the expansion of *R. longirostre* towards the north-west is climate change [11,41]. In the case of Poland, this has been confirmed by climatological research. For example, compared to 1961–1990, the average annual air temperature in 2011–2020 increased from 7.5 °C to 9.1 °C, while the average winter temperature increased from –1.9 °C to −0.2 °C [42]. Another factor undoubtedly assisting the spread of this species in Poland is that roadside verges are being mown less often. This allows hollyhocks to grow to their full extent, flower and bear fruit, creating opportunities for these insects to complete their development.

## Figures and Tables

**Table 1:** Measurements of body parts of mature larvae and pupae of *Rhopalapion longirostre*. BL, body length; BW, body width; HH, head height; HW, head width; PW, pronotum width; RL, rostrum length. All measurements are in millimetres (mm).

	Male Pupa				Female Pupa				Larva			
No.	BW	BL	RL	PW	BW	BL	RL	PW	BL	BW	HH	HW
1	1.15	3.25	1.05	0.75	1.1	3	1.75	0.75	3.25	1.35	0.55	0.6
2	0.75	2.7	0.65	0.75	1.15	2.75	2.05	0.7	3.35	1.1	0.55	0.6
3	1.05	2.75	1	0.75	1.25	2.8	2.25	0.7	3.4	1.6	0.6	0.65
4	1.15	2.65	1	0.75	1.1	2.75	2	0.7	3.2	1.2	0.55	0.6
5	1.3	3.05	0.9	0.8	1	3	2.25	0.7	3.5	1.1	0.5	0.55
6	1.2	3	0.95	0.65	0.8	3	1.1	0.75	3.3	1.2	0.6	0.65
7	1.05	3	0.85	0.7	0.95	2.3	1.1	0.6	3.05	1.15	0.55	0.6
8	0.6	3	0.65	0.5	1.05	3	1.75	0.75	3.85	1.25	0.6	0.675
9	0.95	3	0.65	0.55	1.25	3.35	1.8	0.75	3.65	1.35	0.575	0.625
10	1	2.55	0.75	0.55	1.35	3.65	1.85	0.7	3.9	1.5	0.55	0.6
11	1.3	3.5	1.15	0.85	1.35	3.35	2.15	0.75	3.7	1.4	0.6	0.65
12	1.25	3	0.9	0.7	1.2	3.25	1.8	0.9	4.1	1.25	0.6	0.65
13	0.7	1.75	0.65	0.45	1.35	3.25	2	0.8	4.05	1.6	0.525	0.575
14	1.1	3	0.9	0.7	1.1	3	2.1	0.8	4	1.45	0.575	0.625
15	1.15	3.35	0.85	0.65	1.45	3	2.1	0.75	3	1.35	0.55	0.6
16	1.3	3.15	1.1	0.75	1.3	3.25	2.15	0.75	4.2	1.75	0.625	0.655
17	1.25	2.9	1.2	0.75	1.25	3.4	2.3	0.75	3.2	1.3	0.55	0.6
18	1.2	2.95	1	0.75	1.3	3.35	2.15	0.85	3.9	1.6	0.56	0.61
19	1.2	2.25	1.1	0.7	1.3	3.3	2.15	0.8	3.5	1.35	0.625	0.66
20	1	2.3	0.9	0.6	1.2	3.2	1.95	0.8	3.5	1.2	0.55	0.6
21	1.25	1.8	0.65	0.45	1.1	2.85	1.8	0.85	3.7	1.6	0.55	0.6
Min	0.6	1.75	0.65	0.45	0.8	2.3	1.1	0.6	3	1.1	0.5	0.55
Max	1.3	3.5	1.2	0.85	1.45	3.65	2.3	0.9	4.2	1.75	0.625	0.675
Median	1.15	3	0.9	0.7	1.2	3	2	0.75	3.5	1.35	0.55	0.6
Mean	1.09	2.80	0.90	0.67	1.19	3.09	1.93	0.76	3.59	1.36	0.57	0.62

## Data Availability

All relevant data are within the paper.
